# Prognostic value of a cell cycle progression signature for prostate cancer death in a conservatively managed needle biopsy cohort

**DOI:** 10.1038/bjc.2012.39

**Published:** 2012-02-23

**Authors:** J Cuzick, D M Berney, G Fisher, D Mesher, H Møller, J E Reid, M Perry, J Park, A Younus, A Gutin, C S Foster, P Scardino, J S Lanchbury, S Stone

**Affiliations:** 1 Centre for Cancer Prevention, Wolfson Institute of Preventive Medicine, Queen Mary, University of London, Charterhouse Square, London EC1 M 6BQ, UK; 2 Department of Molecular Oncology, Barts Cancer Institute, Queen Mary, University of London, London EC1 M 6BQ, UK; 3 King's College London, Thames Cancer Registry, London, SE1 3QD, UK; 4 Myriad Genetics, Inc., 320 Wakara Way, Salt Lake City, UT 84108, USA; 5 Department of Cellular Pathology and Molecular Genetics, Liverpool University, Liverpool, L1 3GA, UK; 6 Department of Urology, Memorial Sloan-Kettering Cancer Center, 1275 York Avenue, New York, NY 10021, USA

**Keywords:** localised prostate cancer, prognostic factors, cell cycle genes, expression profiles, CCP score, needle biopsy

## Abstract

**Background:**

The natural history of prostate cancer is highly variable and it is difficult to predict. We showed previously that a cell cycle progression (CCP) score was a robust predictor of outcome in a conservatively managed cohort diagnosed by transurethral resection of the prostate. A greater need is to predict outcome in patients diagnosed by needle biopsy.

**Methods:**

Total RNA was extracted from paraffin specimens. A CCP score was calculated from expression levels of 31 genes. Clinical variables consisted of centrally re-reviewed Gleason score, baseline prostate-specific antigen level, age, clinical stage, and extent of disease. The primary endpoint was death from prostate cancer.

**Results:**

In univariate analysis (*n*=349), the hazard ratio (HR) for death from prostate cancer was 2.02 (95% CI (1.62, 2.53), *P*<10^−9^) for a one-unit increase in CCP score. The CCP score was only weakly correlated with standard prognostic factors and in a multivariate analysis, CCP score dominated (HR for one-unit increase=1.65, 95% CI (1.31, 2.09), *P*=3 × 10^−5^), with Gleason score (*P*=5 × 10^−4^) and prostate-specific antigen (PSA) (*P*=0.017) providing significant additional contributions.

**Conclusion:**

For conservatively managed patients, the CCP score is the strongest independent predictor of cancer death outcome yet described and may prove valuable in managing clinically localised prostate cancer.

Prostate cancer is now the most common cancer in men in the developed world, especially when prostate-specific antigen (PSA) testing is used (Jemal *et al*, 2009; Ferlay *et al*, 2010). Its natural history is highly variable and difficult to predict. Some men have indolent disease that can be safely followed without immediate treatment, whereas others have an aggressive cancer and benefit from immediate intervention. Accurate prediction of disease behaviour is critical because radical treatment is associated with high morbidity. The problems associated with an uncertain prognosis have been exacerbated by the introduction of PSA testing in some countries, leading to an increase in reported incidence but having at most a small effect on mortality rates (Lu-Yao and Greenberg, 1994; Chou *et al*, 2011). Autopsy series indicate a very high prevalence of asymptomatic prostate cancer in 70-year-old men dying from other causes (28% (Breslow *et al*, 1977), 25% (Billis, 1986), 64% (Sakr *et al*, 1994), 33% (Sánchez-Chapado *et al*, 2003), 50% (Soos *et al*, 2005)), which is more than five times higher than the lifetime risk of dying from prostate cancer in the western world, indicating that intensive screening increases the detection of indolent disease (Brawley, 1997; Etzioni *et al*, 2002).

Clinical variables including Gleason score, tumour stage, and PSA concentration have been used at the time of diagnosis to predict disease outcome. However, predictions based on these variables are far from perfect, leading to considerable uncertainty among physicians and patients about the best course for initial treatment. In a recent study, we showed that a cell cycle progression (CCP) score added a substantial amount of prognostic information regarding death from prostate cancer in a cohort of men with clinically localised disease diagnosed by transurethral resection of the prostate (TURP) and managed conservatively (Cuzick *et al*, 2011). Similar results were also seen for biochemical progression in men who were treated by radical prostatectomy.

However, to make a significant impact in the clinic, prognostic markers such as the CCP score, must demonstrate clinical utility when generated from diagnostic needle biopsies. In this study, we report on the ability of the CCP score to predict death from prostate cancer, when measured in needle biopsy material, in a cohort of men with clinically localised disease diagnosed by a needle biopsy and managed conservatively.

## Patients and methods

### Patients

Potential cases of adenocarcinoma of the prostate were identified from six cancer registries in Great Britain. Case notes from collaborating hospitals were reviewed, and full details of these patients have been reported (Cuzick *et al*, 2006, 2011). Men were included in this study if they had conservatively treated clinically localised prostate cancer, which was diagnosed by use of needle biopsy between 1990 and 1996 (inclusively), were younger than 76 years at the time of diagnosis, and had a baseline PSA measurement. Patients treated with radical prostatectomy or radiation therapy, within the first 6 months after diagnosis, or who died or showed evidence of metastatic disease within 6 months of diagnosis were excluded. Men who had hormone therapy before the diagnostic biopsy were also excluded. Original histological specimens from the diagnostic procedure were requested, collected, and centrally reviewed by a panel of expert urological pathologists to confirm the diagnosis of adenocarcinoma and, where necessary, to reassign Gleason scores by use of a contemporary and consistent interpretation of the Gleason scoring system (Montironi *et al*, 2005). Follow-up was conducted through the cancer registries and the last review took place in December 2006. For the purpose of establishing study maturity, follow-up was computed as potential follow-up, commencing at date of diagnosis and is reported for all patients as if alive at the end of the study. Deaths were divided into those from prostate cancer and those from other causes, according to World Health Organization standardised criteria (WHO, 2010). National ethics approval was obtained from the Northern Multicentre Research Ethics Committee, followed by local ethics committee approval at each of the collaborating hospitals.

### Sample preparation and real-time PCR

Approximately 2–4 mm lengths of tumour, identified by review of the corresponding H&E section, were excised from the needle biopsy blocks using scalpel blades spaced at 4 and 2 mm, as previously described (Jhavar *et al*, 2005). The amount dissected was determined both by the length of cancer available in the core, and also to enable preservation of any remaining cancer tissue for tissue micro array studies. Paraffin was removed by xylene treatment and the tumour sample was washed with ethanol. Samples were then digested overnight with proteinase K digestion at 55°C.

Total RNA was extracted with miRNeasy (Qiagen, Valencia, CA, USA) as described by the manufacturer (with the exception of the extended proteinase K digestion).

Total RNA was treated with DNase I (Sigma-Aldrich, St Louis, MO, USA) before cDNA synthesis. We used the High-capacity cDNA Archive Kit (Applied Biosystems, Foster City, CA, USA) to convert total RNA into single-strand cDNA as described by the manufacturer. Ideally, at least 200 ng of RNA was required for the reverse transcription, but use of smaller input amounts was also successful. The quality of the RNA was not ideal because of sample age. To generate a CCP score, essentially all 15 housekeeping genes and at least 21 of the 31 CCP genes had to be amplified. We attempted to generate a CCP score from every sample. For some of the samples, some genes did not amplify, indicating that the RNA quality was too poor to obtain a score. Before measurement of gene expression, the cDNA was preamplified in a pooled reaction containing TaqMan assays (Applied Biosystems). Preamplification reaction conditions were 14 cycles at 95°C for 15 s and 60°C for 4 min. The first cycle also included 10 min incubation at 95°C. The amplification reaction was diluted 1 : 20 with the 1 × Tris-EDTA buffer before it was loaded on TaqMan Low Density arrays (Applied Biosystems) to assess the amplified genes. Expression data were recorded as a threshold cycle value, the PCR cycle at which the fluorescence intensity exceeded a predefined threshold.

### Cell cycle progression score

We attempted to compute the CCP score for each individual, where adequate material was available. A total of 31 predefined CCP genes and 15 housekeeper genes were amplified on one TaqMan Low Density array. Full details have been published (Cuzick *et al*, 2011) and also given in Supplementary Tables ST2 and ST3. The values of each of three replicates of each of the 31 CCP genes were normalised by subtraction of the average of up to 15 non-failed housekeeper genes for that replicate.

### Statistical analysis

Survival analysis was carried out with a Cox proportional hazards model. The primary endpoint was time to death from prostate cancer. Observations were censored on the date of last follow-up, or at death from other causes. Covariates evaluated were: centrally reviewed Gleason primary grade and score, baseline PSA value, clinical stage, extent of disease (proportion of positive cores), age at diagnosis, Ki-67 immunohistochemistry, and initial treatment (no initial treatment or early hormone management).

All PSA values after treatment with hormones or orchiectomy or within 3 weeks after a surgical procedure to the prostate were excluded. Baseline PSA concentration was defined as the last prediagnostic PSA measurement within 6 months before diagnosis. If no such PSA value was available, we took the first post-diagnostic PSA within 6 months; failing that, the prediagnostic PSA taken closest to the date of diagnosis was used. The analysis set and a complete analysis plan were pre specified and all CCP scores were assigned, before the clinical and outcome data were unmasked.

The concentration of PSA was modelled as the natural logarithm of (1+PSA (ng ml^−1^)). Patients with PSA values greater than 100 ng ml^−1^ were excluded as likely to be metastatic disease. For simplicity, Gleason scores were grouped into less than 7, equal to 7, and greater than 7. Little difference was seen between Gleason 3+4 and 4+3, so they were combined (Cuzick *et al*, 2006).

All *P*-values were two-sided and 95% CIs and *P*-values were based on *χ*^2^ statistics with 1 degree of freedom, unless otherwise indicated, obtained from partial likelihoods of proportional hazards models.

The main assessment was a univariate analysis of the association between death from prostate cancer and CCP score. A further predefined assessment of the added prognostic information after adjustment for the baseline variables was also undertaken. This later effect was measured by use of the decrease in the likelihood ratio *χ*^2^, when the CCP score was omitted from a model containing it and the other relevant baseline clinicopathological variables. A multivariate Cox proportional hazards model was used for this purpose and to create a combined score based on the major clinical variables and the CCP score. For the primary analysis, the CCP score was evaluated as a linear term. We used a forward stepwise regression in which a new variable was added only if it had a *P-*value of less than 0.05. Exploratory analyses included adding a quadratic term for the CCP score to further evaluate the shape of the dose-response curve for predicting death from prostate cancer, testing for proportional hazards, evaluating predictive value in years 0–5 and 5+ separately, and testing for interactions of the CCP score with individual covariates. Statistical analyses were carried out with STATA (version 11.2; StataCorp, College Station, TX, USA) and R (version 2.12 ; The R Foundation for Statistical Computing, Vienna, Austria, http://www.R-project.org).

## Results

The assembly of the cohort is shown in the Supplementary Figure SF1. Out of 776 patients diagnosed by needle biopsy and for which a section was available to review histology, needle biopsies were retrieved from 527, and of them, 442 had adequate biopsy material to assay. Of these, 349 (79%) produced a CCP score and had complete baseline and follow-up information. The median potential follow-up time was 11.8 years. A total of 90 deaths from prostate cancer occurred within the 2799 person-years of actual follow-up. The demographic and tumour characteristics of these men are shown in Supplementary Table ST1 and compared with the entire needle cohort. No factor was significantly different at the 1% level, indicating that this was a representative sample.

**Figure 1:**
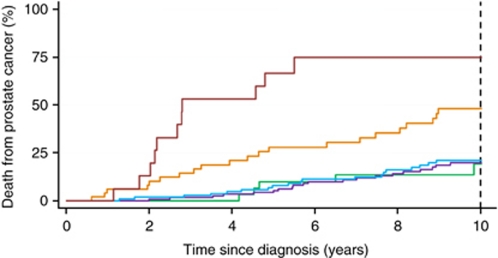
Kaplan–Meier estimates of prostate cancer death according to CCP score. Different categories of CCP score are shown by different coloured lines: red, CCP score>3 (*n*=16), orange, 2<CCP score ⩽3 (*n*=50); blue, 1<CCP score ⩽2 (*n*=114); purple, 0<CCP score ⩽1 (*n*=133); green, CCP score ⩽0 (*n*=36).

The median CCP score was 1.03 and the interquartile range was from 0.41 to 1.74. In the primary univariate analyses, a one-unit increase in CCP score was associated with a 2.02-fold increase in the hazard of dying from prostate cancer. This was highly significant (*χ*^2^=37.6, *P*=8.6 × 10^−10^, 95% CI (1.62, 2.53)). This corresponds to a hazard ratio (HR) of 2.56 (95% CI 1.90, 3.45), for a change from the 25th to 75th percentile of the CCP score distribution (Table 1). The 10-year death rate from prostate cancer for one-unit groups of the CCP score is shown in Figure 1. For those with a score less than 0, the rate was 19.3% and increased to 19.8%, 21.1%, 48.2%, and 74.9% for CCP score groups (0–1, 1–2, 2–3, and >3, respectively). Assuming a linear relationship, the 10-year death rate from prostate cancer, estimated from CCP score alone, is shown in Supplementary Figure SF2. The CCP score was only weakly correlated with other known prognostic factors; Gleason: *r*=0.37; PSA: *r*=0.14; extent of disease: *r*=0.28.

**Figure 2:**
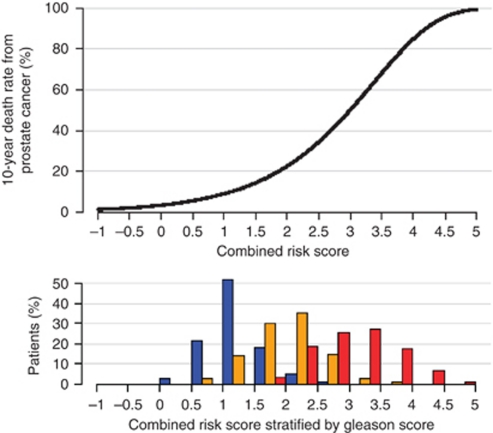
Ten-year predicted risk of prostate cancer death according to combined risk score, and a histogram of the combined score in different Gleason score categories. Different categories are shown by different coloured bars: blue, Gleason score <7; orange, Gleason score=7; red, Gleason score >7.

In the pre-planned multivariate analyses, extent of disease, age, clinical stage, and use of hormones were not statistically significant and only CCP score, Gleason grade, and PSA remained in the final model (Table 1). Ki67 was included in the multivariate analysis, but was not significant (results not shown). The HR for a one-unit change in the CCP score in the multivariate model was 1.65 (95% CI 1.31, 2.09) *χ*^2^=17.7, *P*=3 × 10^−5^), and it was a stronger prognostic factor than either Gleason grade or PSA. This corresponds to a HR of 1.96 (95% CI 1.43, 2.68), for a change from the 25th to 75th percentile of the CCP score distribution. The best linear predictor combining these variables was 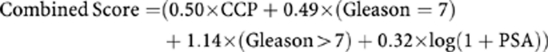 In exploratory analyses, we found that adding a quadratic term for the CCP score in the multivariate analysis was statistically significant (*P*=0.008) and with this extra term, CCP score was even more significant (*χ*^2^ (2.d.f)=24.8, *P*=4 × 10^−6^) than CCP score alone (*P*=3 × 10^−5^). There was also evidence of a strong effect of the CCP score in predicting death in the first 5 years of follow-up (multivariate HR=2.14, (95% CI 1.55, 2.95) *χ*^2^=22, *P*=3 × 10^−6^) with a much lesser effect thereafter (multivariate HR=1.27, (95% CI 0.92, 1.75) *χ*^2^=2.1, *P*=0.15). Both of these observations can be explained by the strong effect of high CCP score on early deaths. Further data are needed to confirm these observations, regarding the apparent super-linear form of the prognostic value of the CCP score and the duration of its impact. The 10-year risk of death from prostate cancer across the range of this combined score is shown in Figure 2.

**Figure 3:**
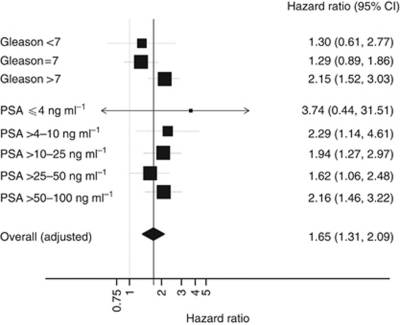
Hazard ratio for prostate cancer mortality for a one-unit change in CCP score for different clinical subgroups. The area of the box is proportional to number of events in each group, and the horizontal bars represent 95% confidence intervals (CI).

**Figure 4:**
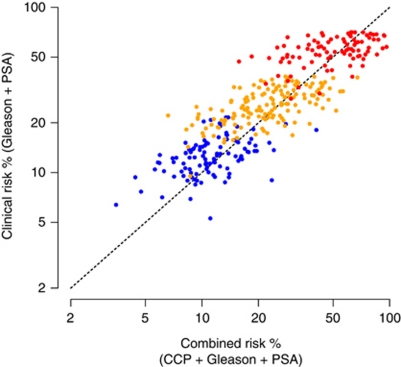
Scatter plot of predicted 10-year risk of death from prostate cancer for combined risk score *vs* clinical risk score. Different Gleason score categories are shown by different coloured dots: (blue, Gleason < 7; orange, Gleason=7; red, Gleason >7), whereas the vertical axis indicates the added information in PSA and the horizontal axis for PSA and CCP score. For any given patient, the added contribution of the CCP score to the predicted risk, based on Gleason and PSA, can be determined by the horizontal distance between the dot and the diagonal dashed line.

A forest plot of the prognostic value of the CCP score by Gleason grade and PSA level is shown in Figure 3. Little variation was seen except for a non-significant trend toward higher HRs for the CCP score in higher Gleason grades (*P*-value for heterogeneity=0.13, Q Statistic). To illustrate how the addition of CCP score changes patient prognosis, we compared the prediction of 10-year prostate cancer death rate based on the combined score to the prediction obtained from using only PSA and Gleason score (Figure 4). A useful discrimination is seen within each Gleason score, and adding both CCP score and PSA was seen to provide more discrimination than adding PSA alone.

## Discussion

These results confirm our previous findings (Cuzick *et al*, 2011) on the prognostic value of the CCP score measured after radical surgery and in TURP specimens, and extend them to the clinical situation where the CCP score is generated from tissue obtained from diagnostic needle biopsies. In this clinical setting, CCP score was highly prognostic, and provided more information than either Gleason score or PSA. In addition, because it was only weakly correlated with other clinical variables it provides important independent information that cannot be obtained otherwise.

Although this cohort does not reflect contemporary treatment of prostate cancer, in that a much greater proportion of patients were managed conservatively, it does have the advantage of providing information on the cancer death rates in this wider population and thus facilitates the ability to identify which patients will do well or poorly with conservative management in a broader context than would be possible with contemporary cohorts. To the best of our knowledge, this is the first study in any cancer to evaluate the prognostic value of an mRNA-based test in needle biopsies. Needle biopsies sample only a small portion of the tumour and provide a limited amount of tissue from which to generate molecular data. Both limitations, tumour sampling and tissue amount, could have adversely affected the ability of this molecular assay to predict disease outcome at diagnosis. However, we found that CCP score generated from needle biopsies predicted prostate cancer death more accurately than any other known factor and that 80% of biopsies provided enough material to generate an acceptable molecular signature, despite the fact that the material was formalin fixed and more than 10 years old. For this study, 2–4 mm linear segments of tumour material were excised from the paraffin-embedded biopsy, which is not a routine procedure. Ongoing studies are using up to ten multiple sections from the tumour material. Based on limited experience, we have found that this procedure in contemporary biopsy specimens yields enough quality RNA to generate acceptable test results, but more empirical data are needed.

Based on exploratory analyses presented here, there is evidence that the CCP score may have a non-linear impact on the predicted probability of prostate cancer death. This could either be due to a true non-linear relationship between the CCP score and risk of death from prostate cancer, or a lack of proportional hazards in that the CCP score is a better predictor of earlier deaths. Although the data set is not large enough to distinguish between these two possibilities, we think that the latter is likely to be at least a partial explanation for two reasons. First, time-dependent effects are commonly seen with prognostic markers (Fentiman *et al*, 1984). Second, we see evidence for time dependence for CCP score in this study and in a retrospective analysis of our TURP cohort (data not shown). If confirmed, these data suggest it may be useful to retest cancer found in subsequent needle biopsy specimens, especially if other factors such as PSA suggest progression, to determine appropriate management. Unfortunately, no follow-up biopsy specimens were available for this study.

Misclassification of cause of death is a concern, but this will generally weaken any real association of CCP score with prostate cancer death. As a check, we evaluated the predictive value of the risk factors, considered in this paper, for death from other causes (censoring at death from prostate cancer). Only age was a strong predictor, and none of the known factors for prostate cancer death nor CCP score were significant at the 1% level.

Here, in contrast to our previous studies, we see a trend towards a larger effect in patients with Gleason score greater than 7. Although this trend was not significant, if confirmed, it suggests that the CCP score may help stratify high-risk localised prostate cancers into a favourable group that could be treated with local therapy only *vs* an unfavourable group, more likely to develop metastases quickly, whose survival could be improved with early adjuvant systemic chemohormonal therapy, a question under study in current clinical trials (e.g. NCT00430183).

The most obvious clinical use of the CCP score is to help identify low-risk patients who can be safely managed by surveillance. In this series, we were unable to identify a clinically significant subgroup with a 10-year risk of dying from prostate cancer of less than 5%. However, the CCP score increased the ability to identify men with a less than 10% risk of dying from prostate cancer within 10 years, from 7 to 14%. In addition, for patients with a Gleason score of 6, where considerable uncertainty still exists as to appropriate treatment, the predicted 10-year prostate cancer death rate with the addition of the CCP score ranged from 3.5 to 41.0% (compared with 5.1 to 20.9% using clinical parameters only). We believe this is relevant information when considering appropriate care. However, as deaths from prostate cancer are rare in this group, larger cohorts are needed to fully characterise the value of the CCP score in identifying very low-risk patients, and a clearer relationship may emerge when more patients have been studied.

## Figures and Tables

**Table 1:** Univariate and multivariate analysis for death from prostate cancer

	**Univariate analysis**				**Multivariate analysis**			
**Variable**	***N***	***χ*^2^** **(1 df)**	**Hazard ratio (95% CI)**	***P*-value**	***N***	***χ*^2^ (1 df)**	**Hazard ratio^a^ (95% CI)**	***P-*value**
CCP score	349	37.6	2.02 (1.62, 2.53)	8.6 × 10^−10^	349	17.7	1.65 (1.31, 2.09)	2.6 × 10^−5^
*Gleason score*								
<7	106	36.4	0.46 (0.25, 0.86)	1.6 × 10^−9^	106	12.1	0.61 (0.32, 1.16)	5.0 × 10^−4^
7	152		1 (ref)		152		1 (ref)	
>7	91		2.70 (1.72, 4.23)		91		1.90 (1.18, 3.07)	
log (1+PSA) (ng ml^−1^)	349	16.8	1.70 (1.31, 2.20)	4.2 × 10^−5^	349	5.7	1.37 (1.05, 1.79)	0.017
*Extent of disease* b								
<50%	69	14.1	0.50 (0.22, 1.12)	0.0002				
50–<100%	106		1 (ref)					
100%	160		1.66 (1.01, 2.73)					
								
Age at diagnosis (years)	349	0.05	1.00 (0.96, 1.04)	0.82				
*Clinical stage*								
T1	38	3.72	0.75 (0.32, 1.75)	0.054				
T2	106		1 (ref)					
T3	43		1.74 (0.90, 3.38)					
*Hormone use*								
No	200	10.2	1 (ref)	0.001				
Yes	149		1.97 (1.30, 2.98)					

Abbreviations: χ^2^=chi-square; df=degrees of freedom; CI=confidence interval; ref=reference category; PSA=prostate-specific antigen.

a Gleason score assessed with 2df for computing the hazard ratios in the multivariate analysis.

b Proportion of positive cores.
