# Supplementary material for: Prognostic value of a cell cycle progression signature for prostate cancer death in a conservatively managed needle biopsy cohort
Source: Br J Cancer. 2012 Feb 23;106(6):1095–9. doi: 10.1038/bjc.2012.39 (PMC3304411; doi:10.1038/bjc.2012.39)
Supplement: Supplementary Information [file bjc201239x1.doc]

**Supplementary online material:**

| **Supplementary Table ST1: Demographics of Needle Biopsies** | | | |  |
| --- | --- | --- | --- | --- |
|  |  |  | | |
|  | Selected Needle Biopsies (n=349) | Remaining Needle* Biopsies (n=425) | |  |
| Follow-up (years) | 11.8 (10.8, 12.7) | 11.7 (10.9, 12.7) | |  |
| Age (years) | 70.5 (65.8, 73.4) | 70.3 (66.8, 73.0) | |  |
| Gleason Score |  |  | |  |
| <7 | 106 (30.4) | 172 (40.5) | |  |
| 7 | 152 (43.6) | 138 (32.5) | |  |
| >7 | 91 (26.1) | 115 (27.1) | |  |
| PSA (ng/ml) | 21.4 (11.9, 42.0) | 19.5 (10.3, 38.0) | |  |
| 10-year death from prostate cancer | 27.1 (21.6, 32.2) | 27.7 (23.2, 32.9) | |  |
| 10-year death from any cause | 54.4 (48.9, 59.4) | 57.6 (52.9, 62.3) | |  |
| Numbers are median (IQR) or n (%) as appropriate | | |  |  |
| * Patients with missing baseline (reviewed Gleason and PSA) values (n=2) were omitted | | |  |  |

Abbreviations: PSA, prostate specific antigen

Table ST2 Complete list of evaluated 31 CCP genes

|  |  |
| --- | --- |
| Gene name | Correlation with CCP mean |
| FOXM1 | 0.908 |
| CDC20 | 0.907 |
| CDKN3 | 0.9 |
| CDC2 | 0.899 |
| KIF11 | 0.898 |
| KIAA0101 | 0.89 |
| NUSAP1 | 0.887 |
| CENPF | 0.882 |
| ASPM | 0.879 |
| BUB1B | 0.879 |
| RRM2 | 0.876 |
| DLGAP5 | 0.875 |
| BIRC5 | 0.864 |
| KIF20A | 0.86 |
| PLK1 | 0.86 |
| TOP2A | 0.851 |
| TK1 | 0.837 |
| PBK | 0.831 |
| ASF1B | 0.827 |
| C18orf24 | 0.817 |
| RAD54L | 0.816 |
| PTTG1 | 0.814 |
| CDCA3 | 0.811 |
| MCM10 | 0.802 |
| PRC1 | 0.79 |
| DTL | 0.788 |
| CEP55 | 0.787 |
| RAD51 | 0.783 |
| CENPM | 0.781 |
| CDCA8 | 0.774 |
| ORC6L | 0.736 |

| Table ST3 List of 15 housekeeping genes | |  |
| --- | --- | --- |
|  |  |  |
| Gene | Correlation with HK Mean | |
|  |  | |
| RPL38 | 0.989 | |
| UBA52 | 0.986 | |
| PSMC1 | 0.985 | |
| RPL4 | 0.984 | |
| RPL37 | 0.983 | |
| RPS29 | 0.983 | |
| SLC25A3 | 0.982 | |
| CLTC | 0.981 | |
| TXNL1 | 0.98 | |
| PSMA1 | 0.98 | |
| RPL8 | 0.98 | |
| MMADHC | 0.979 | |
| RPL13A;LOC728658 | 0.979 | |
| PPP2CA | 0.978 | |
| MRFAP1 | 0.978 | |

Supplementary Figure SF1: Consort diagram - overview of needle cohort.


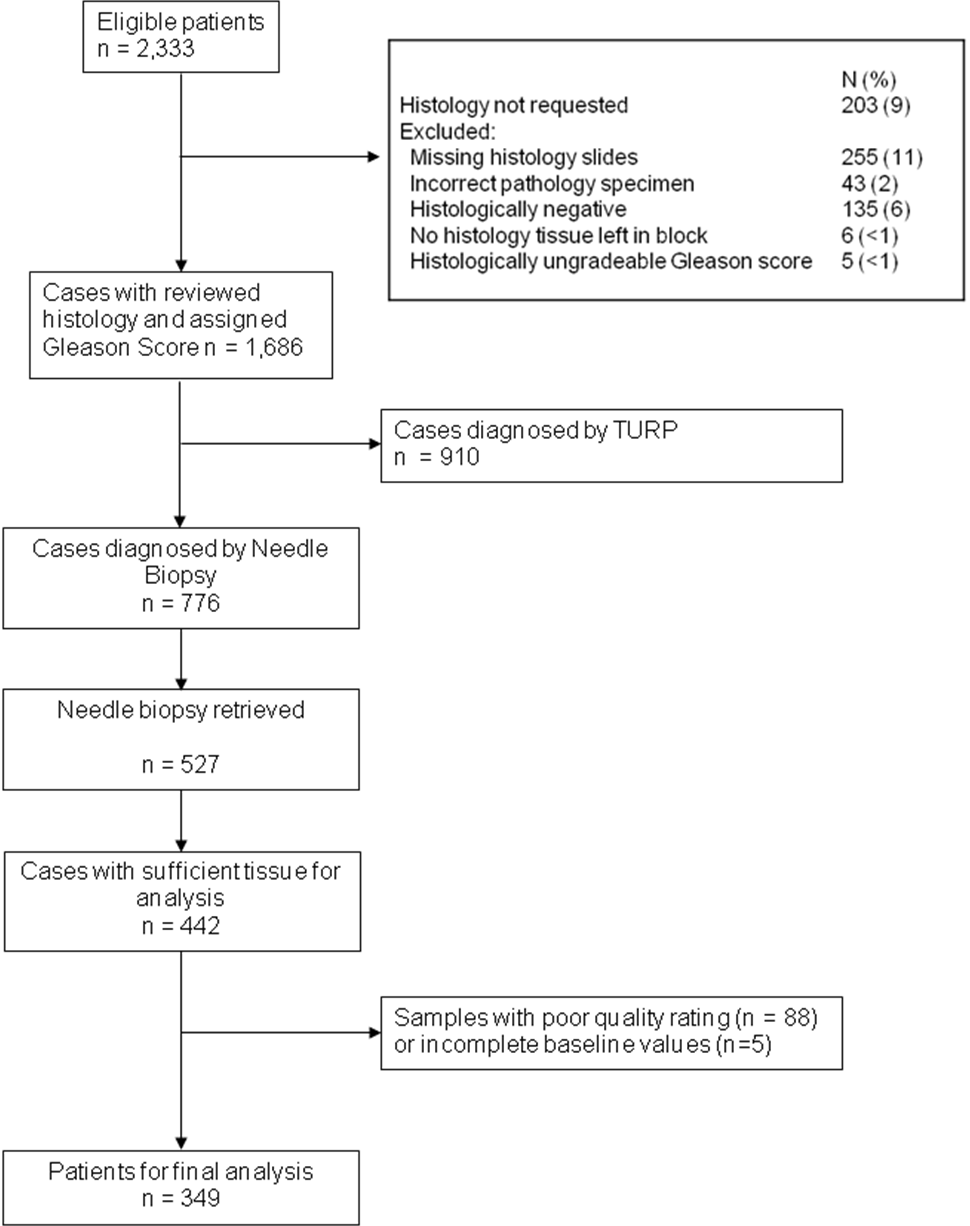


Supplementary Figure SF2: Assuming a linear relationship, the 10-year death rate from prostate cancer for different values of the CCP score and a histogram of the CCP score. Quartiles of the CCP score are denoted by Q1 Q2 Q3.

Appendix

Investigators in participating regional cancer registries, research centres and hospital trusts are listed below. Members of the Transatlantic Prostate Group are designated by an asterisk.

Thames Cancer Registry: Henrik Møller^*^, Shirley Bell (deceased), K. Linklater, J. Ottey V. Fisher; Ashford & St. Peter’s, M. Hall, N. Harvey Hills; Barnet & Chase Farm, H. Reid; Brighton and Sussex, N. Kirkham, P. Thomas; Bromley, D. Nurse; Dartford & Gravesham, I. Dickinson, P. Thebe; East & North Hertfordshire, D. Hanbury, M. Ali-Izzi; Eastbourne, C. Moffatt; Epsom & St. Helier, M. Bailey, L. Temple; Essex Rivers Healthcare, W. Aung, C. Booth; Frimley Park, B. Montgomery, P. Denham; Greenwich Healthcare, N. Cetti, P. Pinto; Guy’s & St Thomas’s, A. Chandra, T. O’Brien; Hammersmith Hospitals, N. Livni; Havering Hospitals, I. Saeed; Hillingdon, F. Barker, T. Beaven; King’s Healthcare, G. Muir, Z. Khan; Kingston, C. Jameson; Lewisham, A. Giles; Mayday Healthcare, N. Arsanious, A. Arnaout; The Medway, E. Boye; Mid Essex Hospitals*;* Mid Kent, M. Boyle; North West London Hospitals, M. Jarmulowicz,; Royal Free Hampstead, R. J. Morgan, A. Bates; St Bartholomew’s and The Royal London Hospitals, F. Chinegwundoh, R. T. D. Oliver*, D. Berney*; Wolfson Institute of Preventive Medicine, Queen Mary University of London, J Cuzick*, G.Fisher*, L.Ambroisine*; Institute of Cancer Research, Sutton, C Cooper*; Royal Surrey County, S. De Sanctis; Southend, M. Chappell; St George’s, London, R. Kirby, C. Corbishley; St Mary’s, London, A. Patel, M. Walker; West Hertfordshire, J. Crisp, W. Riddle; Worthing & Southlands Hospitals, J. Grant.

Northern & Yorkshire Cancer Registry & Information Service: David Forman^*^, C. Storer, C. Bennett, C. Spink; Airedale, I. Appleyard, J. O’Dowd; Hull & East Yorkshire, J. Hetherington, A. MacDonald; The Leeds Teaching Hospitals, P. Whelan, P. Quirke, P. Harnden.

Oxford Cancer Intelligence Unit: Monica Roche^*^, Sandra Edwards, S. Bose, P. Hall; Heatherwood & Wexham Park, M. Ali, O. Karim; Milton Keynes, E. Walker, S. Jalloh; Northampton, M. Miller, A. Molyneux; Oxford Radcliffe, S. Brewster, D. Davies; Royal Berkshire & Battle, P. Malone, C. M^c^Cormick; Stoke Mandeville, J. Greenland, A. Padel

Welsh Cancer Intelligence & Surveillance Unit: John Steward^*^, Shelagh Reynolds, Lynda Roberts, Judith Adams; Ceredigion and Mid Wales, J. Edwards, C.G.B. Simpson; Conwy & Denbighshire, A. Dalton, V. Srinivasan; NE Wales, A. De Bolla, C. Burdge; Gwent Healthcare, W. Bowsher, M. Rashid; Swansea, M. Lucas, C. O’Brien; Cardiff & Vale, M. Varma.

Scottish Cancer Registry: David Brewster^*^; The Lothian University Hospitals, J.Royle, K.Grigor; North Glasgow University Hospitals, D.Kirk, A Milano, R.Reid.

Merseyside & Cheshire Cancer Registry: Lyn Williams^*^, R. Iddenden; Royal Liverpool University Hospital, C.S. Foster*, P. Cornford.

Memorial Sloan Kettering Cancer Center: P. Scardino*, P Fearn*, V Reuter*, J Eastham*, M Kattan*, H. Lilja*.
